# Reproducibility of rest and exercise stress contrast-enhanced calf perfusion magnetic resonance imaging in peripheral arterial disease

**DOI:** 10.1186/1532-429X-15-14

**Published:** 2013-01-23

**Authors:** Ronny S Jiji, Amy W Pollak, Frederick H Epstein, Patrick F Antkowiak, Craig H Meyer, Arthur L Weltman, David Lopez, Joseph M DiMaria, Jennifer R Hunter, John M Christopher, Christopher M Kramer

**Affiliations:** 1 Departments of Medicine and the Cardiovascular Imaging Center, University of Virginia Health System, Charlottesville, VA, USA; 2 Departments of Radiology and the Cardiovascular Imaging Center, University of Virginia Health System, Charlottesville, VA, USA; 3 Biomedical Engineering and the Cardiovascular Imaging Center, University of Virginia Health System, Charlottesville, VA, USA; 4 Departments of Medicine and Radiology, University of Virginia Health System, Lee Street, Box 800170, Charlottesville, VA, 22908, USA

## Abstract

**Background:**

The purpose was to determine the reproducibility and utility of rest, exercise, and perfusion reserve (PR) measures by contrast-enhanced (CE) calf perfusion magnetic resonance imaging (MRI) of the calf in normal subjects (NL) and patients with peripheral arterial disease (PAD).

**Methods:**

Eleven PAD patients with claudication (ankle-brachial index 0.67 ±0.14) and 16 age-matched NL underwent symptom-limited CE-MRI using a pedal ergometer. Tissue perfusion and arterial input were measured at rest and peak exercise after injection of 0.1 mM/kg of gadolinium-diethylnetriamine pentaacetic acid (Gd-DTPA). Tissue function (TF) and arterial input function (AIF) measurements were made from the slope of time-intensity curves in muscle and artery, respectively, and normalized to proton density signal to correct for coil inhomogeneity. Perfusion index (PI) = TF/AIF. Perfusion reserve (PR) = exercise TF/ rest TF. Intraclass correlation coefficient (ICC) was calculated from 11 NL and 10 PAD with repeated MRI on a different day.

**Results:**

Resting TF was low in NL and PAD (mean ± SD 0.25 ± 0.18 vs 0.35 ± 0.71, p = 0.59) but reproducible (ICC 0.76). Exercise TF was higher in NL than PAD (5.5 ± 3.2 vs. 3.4 ± 1.6, p = 0.04). Perfusion reserve was similar between groups and highly variable (28.6 ± 19.8 vs. 42.6 ± 41.0, p = 0.26). Exercise TF and PI were reproducible measures (ICC 0.63 and 0.60, respectively).

**Conclusion:**

Although rest measures are reproducible, they are quite low, do not distinguish NL from PAD, and lead to variability in perfusion reserve measures. Exercise TF and PI are the most reproducible MRI perfusion measures in PAD for use in clinical trials.

## Background

There are approximately 8 million patients in the U.S. with PAD [1] which is associated with significant cardiovascular morbidity [2,3]. Traditional methods for evaluating disease severity in PAD include the arterial brachial index (ABI), and anatomical imaging of stenoses such as CT, MR, or x-ray angiography. However, there are no established non-invasive methods available to determine the functional significance of a stenosis or whether increasing macrovascular calf muscle blood flow with bypass grafting, angioplasty, medical therapies, or novel angiogenic approaches improves tissue perfusion.

We have previously demonstrated that first-pass exercise calf perfusion with magnetic resonance imaging (MRI) can distinguish between subjects with PAD and normal controls who performed workload matched to the PAD group as well as exercised maximally [4]. Measures of tissue perfusion correlate with treadmill exercise performance in PAD [5]. Quantification of skeletal muscle flow reserve (the ratio of exercise to rest blood flow) using contrast-enhanced exercise (ultrasound) stress calf perfusion predicts claudication threshold in PAD [6]. Furthermore, calf muscle flow reserve may be a more sensitive method for assessing the contribution of collateralization that occurs in severe PAD [7]. The goal of the present study was to determine the reproducibility of exercise calf perfusion measurements and perfusion reserve using contrast-enhanced MRI in a group of normal subjects and PAD patients.

## Methods

### Study population

We recruited 11 patients with PAD and 16 age-matched normal subjects (NL). The study protocol was approved by the Human Investigation Committee of the Internal Review Board (IRB) at the University of Virginia Health System. All patients signed informed consent prior to participation. Inclusion criteria for PAD patients were age 18–85 and claudication in at least one leg with an ABI >0.4 and <0.9. Exclusion criteria included a glomerular filtration rate <45, critical limb ischemia, conditions other than PAD limiting ability to exercise, peripheral neuropathy, unstable coronary artery disease, decompensated heart failure, and lower extremity vascular surgery or angioplasty within the preceding three months. For assessment of reproducibility, 11 NL and 10 PAD returned on a different day for a repeat study.

With the calf of interest wrapped in a flexible receive coil and centered in the MR scanner, exercise was performed using an MR-compatible pedal ergometer to the timing of a metronome set at a rate of 50 beats per minute. For PAD, the leg with the lower ABI was imaged if both legs were symptomatic and met ABI inclusion criteria. All participants were encouraged to exercise until exhaustion or limiting symptoms. If neither occurred, exercise was discontinued at 20 minutes.

### Magnetic resonance imaging

First-pass calf perfusion MRI images were obtained on a 3 T Trio™ MR scanner (Siemens Healthcare, Erlangen, Germany) both at rest and immediately after cessation of exercise with injection of 0.1 mM/kg of gadolinium-diethylnetriamine pentaacetic acid (Gd-DTPA) (Berlex, Montville, NJ, USA) followed by a 40 mL saline flush administered at a rate of 4 mL/sec through an intravenous (IV) catheter placed in an antecubital vein. A spoiled gradient echo (GRE) dual-contrast sequence as previously described [4] with slices positioned 32 mm apart allowed for simultaneous acquisition of arterial input and tissue perfusion images, the former using saturation recovery (SR) GRE with an inversion time (TI) of 10 ms and the latter using inversion-recovery (IR) GRE with a TI of 300 ms. Additional relevant parameters included slice thickness = 8 mm, number of measurements =150 (75 SR and 75 IR images), field of view =180 mm × 180 mm, image matrix = 64 × 64, repetition time (TR) = 2.8 ms, echo time (TE) = 1.41 ms, excitation flip angle = 15°.The time between successive sets of paired SR and IR images was 1008 ms. Proton density weighted (PDW) images of the corresponding arterial input and tissue perfusion slices were obtained prior to gadolinium administration for subsequent signal normalization to account for any variations in signal attributable to the coil sensitivity profile. PDW images were acquired using the same imaging sequence and parameters as above without applying the saturation or inversion pulses.

### Image analysis

ARGUS image analysis software (Siemens Healthcare) was used for offline data analysis. A circular region of interest with a diameter of approximately 15 mm was placed in several calf muscle groups on the tissue perfusion images with care taken to avoid arteries. A time intensity curve (TIC) was generated for the muscle group with the greatest signal intensity as this was typically the muscle that performed the greatest work with exercise, with the slope of the curve representing the TF. A smaller region of interest was placed on the proximal arterial input slice in the artery that feeds the muscle analyzed above, generally the popliteal artery. The ROI matched the size of the artery. The slope of that TIC represented the AIF. Measurements of the TF and AIF were obtained at rest and immediately after cessation of exercise. Other parameters calculated were PI, defined as TF/AIF, and PR, defined as exercise TF/rest TF. For those NL and PAD patients who underwent a second MRI, analysis was performed using the same muscle group and arterial input for both studies. Studies from 5 normal subjects and 5 PAD subjects were examined by 2 observers (RSJ and DL) to determine interobserver reproducibility.

**Figure 1:**
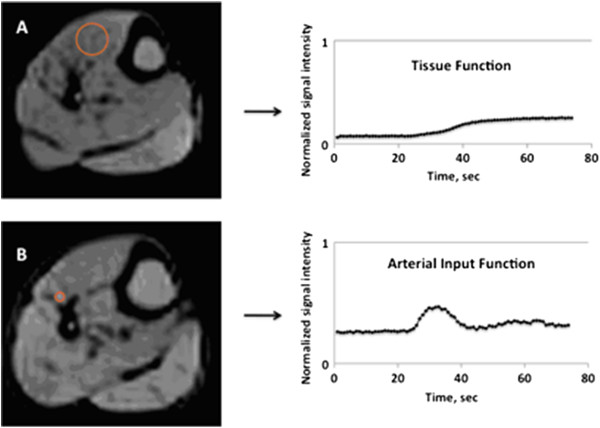
**The tissue function and arterial input function were normalized by proton density to compensate for variations in the receiver coil sensitivity profile. **Examples of region-specific proton density measurements for the tissue function (**A**) and arterial input function (**B**) are shown, as are the corresponding normalized TF and AIF time-intensity curves.

Corresponding regions of interest were placed in the muscle group and feeding artery on PDW images. The arterial input and tissue signal intensities were divided by the proton density measurements to yield normalized arterial input and tissue function curves, with the slope of those curves representing the normalized AIF and TF (Figure 1). 

### Statistical analysis

All statistical analysis was performed using SPSS™ Statistics 20.0 software (IBM). TF, AIF, rest PI, and exercise PI were calculated using both raw and normalized data. Differences in characteristics between NL and PAD patients were evaluated using an unpaired T-test. A two-way intraclass correlation coefficient (ICC) was calculated for the entire cohort (NL + PAD) for all parameters to determine test-retest and interobserver reproducibility. Bland-Altman limits of agreement for exercise perfusion measures were obtained using MedCalc™ software (version 12.2.1).

## Results

Age was similar between NL and PAD (62 ±10 vs. 60 ±12, p = 0.51). The PAD group was predominantly male (91%) as compared to NL (50%), (p = 0.016). Mean ABI was 0.67±0.14 in PAD (Table 1). Amongst the PAD patients, 82% had a history of CAD, 91% had a history of previous or current tobacco use, and 3 patients had prior cerebrovascular accident (CVA). Four PAD patients had undergone previous lower extremity bypass or angioplasty, and the majority were non-diabetic (Table 1). As expected, exercise time was significantly lower for PAD compared to NL (304±320 sec vs. 802±450, p = 0.003). The anterior tibialis (AT) muscle was used for analysis in the majority of NL (75%) and PAD (73%). For two individual MR studies in the PAD group, rest measurements of AIF or TF were unmeasurable due to extremely low rest perfusion. 

**Table 1:** Baseline characteristics of PAD group

**ID**	**Age**	**ABI**	**CAD**	**Tobacco use**	**CVA**	**Diabetes Mellitus**	**Hypertension**	**Prior lower extremityvascular surgery or angioplasty**
1	63	0.83	Y	Prior	Y	N	Y	Y
2	68	0.74	Y	Current	N	Y	Y	Y
3	63	0.56	Y	Prior	N	Y	Y	N
4	60	0.68	Y	Prior	N	Y	Y	N
5	81	0.46	N	N	N	N	Y	N
6	51	0.77	N	Prior	N	N	N	Y
7	45	0.81	Y	Current	N	N	Y	N
8	49	0.52	Y	Current	N	N	Y	Y
9	75	0.59	Y	Prior	Y	Y	Y	N
10	65	0.44	Y	Current	Y	N	Y	N
11	59	0.74	Y	Current	N	N	N	N
Mean ± SD	60 ± 12	0.67 ± 0.14						

**Figure 2:**
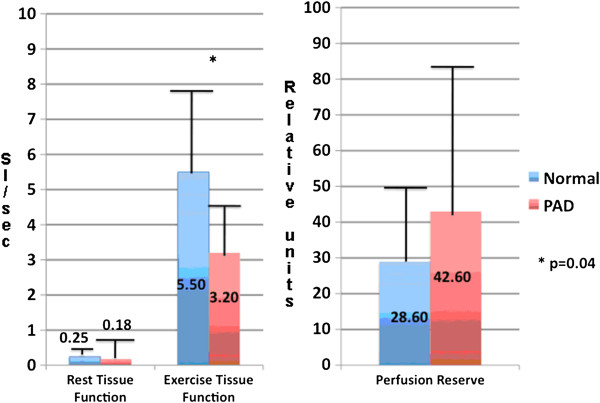
**Rest and exercise tissue function normalized by proton density measurements (left), and perfusion reserve (right) for normal subjects and PAD patients. **The units for tissue function are delta signal intensity/sec.

**Figure 3:**
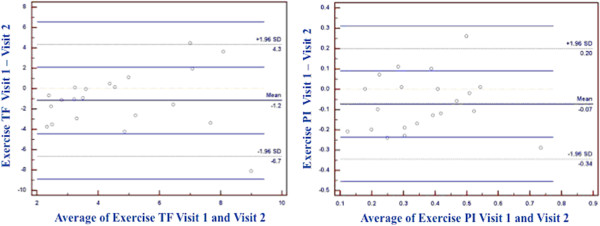
Bland-Altman plots for exercise tissue function (left) and exercise perfusion index (right) including normal and PAD participants.

The mean time between repeated testing was 19 ± 54 days (median 6 days, range 1–256 days). Rest TF and normalized rest TF were the most reproducible (ICC 0.76 and 0.83 respectively) but these measures were quite low and did not differ between NL and PAD (mean ± SD 0.25 ± 0.18 vs 0.35 ± 0.71, p = 0.59) (Figure 2). Signal normalization by proton density greatly improved the reproducibility of exercise TF measures (from 0.35 to 0.63), which distinguished PAD from NL (5.5 ± 3.2 vs. 3.4 ± 1.6, p = 0.04) (Figure 2). Exercise PI was also reproducible (ICC 0.60). Perfusion reserve measurements were reproducible (ICC 0.58), but were quite variable due to the very low rest values in the denominator and thus were unable to distinguish between PAD and NL (28.6 ± 19.8 vs. 42.6 ± 41.0, p = 0.26) (Figure 2). There were two subjects amongst NL and PAD who fell outside of the 95% confidence interval for exercise TF and PI, with Bland Altman limits of agreement −6.66 to 4.34 and −0.34 to 0.20 respectively (Figure 3). All measures demonstrated good interobserver reproducibility, but were better for exercise than rest. The ICC’s for rest AIF, TF, and PI were 0.79, 0.99, and 0.71, respectively. For exercise AIF, TF, and PI, ICC’s were 0.88, 0.98, and 0.86 respectively. 

## Discussion

This study demonstrates that contrast-enhanced exercise calf perfusion MRI measures are reproducible in a cohort of normal subjects and PAD patients. Although rest perfusion measures are highly reproducible, they are less clinically useful as they do not differentiate PAD from normal. Similarly, PR is reproducible, but also less useful as it depends on low rest measures in the denominator which makes PR quite variable. In addition, PR did not differentiate PAD from NL. Signal normalization to proton density improves reproducibility of exercise TF measurements by correcting for variations in signal that may occur due to MR coil placement in relation to the muscle groups being imaged. Exercise TF and PI are reproducible and more likely to be clinically useful as they distinguish between NL and PAD [4]. Interobserver reproducibility was excellent, especially for exercise measures. Thus, for future evaluation of the effects of novel therapies for PAD on muscle perfusion, exercise TF and PI are likely to be the most useful measures.

Our finding that rest perfusion measurements by CE-MRI are reproducible, but extremely low and non-discriminatory suggests decreased sensitivity of rest perfusion by MRI compared to contrast-enhanced ultrasound (CEU) [8]. Time to peak muscle intensity demonstrated by CEU can distinguish degrees of collateralization in PAD patients determined by MR or x-ray angiography [9]. Newer ultrasound techniques such as contrast pulsed sequencing [10] has a spatial resolution of 10–20 μm, and is able to show an increase in resting blood volume and tissue perfusion in preliminary animal studies after exposure to angiogenic factors [11]. Thus, CEU techniques may be an important tool for future investigation of interventions to improve resting tissue perfusion in PAD, where contrast-enhanced MRI approaches may be best used to measure peak exercise and/or hyperemic blood flow. Clinical endpoints currently used to gauge success of medical or surgical interventions in PAD are based on degree of functional impairment (6-minute walk test, claudication time). Therefore, exercise perfusion MR is poised to be a useful and important method to quantify improvement in peak exercise perfusion with novel therapies.

While calf muscle PR has been previously investigated using CEU in PAD patients [6,7], this is the first study to test the reproducibility of MRI measures of PR in both normal subjects and PAD patients. In contrast to CEU, which demonstrates lower perfusion reserve in PAD compared to control subjects [6], CE-MRI was unable to detect a difference, likely due to very low values for rest perfusion in PAD. However, these differing results might also be due to differences in exercise methods (treadmill in the study by Lindner et al.) and the latter study’s inclusion of claudicant PAD patients with an ABI >0.9 [6]. Other methods such as PET have also demonstrated lower PR in PAD subjects and have shown a good correlation with invasive measures of flow reserve [12].

Exercise PI is significantly lower in PAD compared to normal subjects [4], and MRI measurements of exercise TF correlate with the 6-minute walk test in PAD patients [5]. This study demonstrates that exercise PI is a reproducible parameter in NL and PAD. Furthermore, signal normalization using proton density weighted images, which has been shown to correct for inhomogeneities in surface receiver coils in 2D and 3D MR quantitative perfusion analysis of the myocardium [13], improves reproducibility of exercise MR perfusion measurements in the calf.

### Study limitations

The PAD participants were predominantly male and therefore this study cannot draw any conclusions about differences in reproducibility according to gender, although the populations were closely age-matched. Gender may be an important consideration for future research in exercise calf perfusion MRI, as females have a higher prevalence of walking impairment from leg symptoms than males [14].

The potential effects of endothelial dysfunction [15] and increased peripheral resistance [16] on exercise perfusion MR in PAD are not known. Alternatives to exercise such as vasodilation with adenosine [7,12] or cuff-occlusion hyperemia [17] may be more sensitive and reproducible methods to assess changes in both endothelial function and microvascular perfusion in PAD patients. However, PAD patients typically present with exercise-induced symptoms, and poor functional capacity in PAD has been associated with greater all-cause mortality [18]. Thus, exercise remains a more physiologically relevant challenge.

Total workload was not measured during exercise as our group had previously done [4]. Thus, variations in total exercise performed during the two study visits might partly explain the modest reproducibility of some of the exercise MRI calf perfusion measures in the present study. Using different modalities of MR-compatible exercise (pedal ergometer vs. cycle ergometer) also may affect reproducibility.

## Conclusion

We demonstrate that measurements obtained by CE exercise perfusion MRI are reproducible in a cohort of normal subjects and PAD patients. Normalized tissue function and perfusion index measures are both reproducible and discriminatory and therefore likely to be of clinical utility in future studies monitoring the effects of medical, surgical, exercise, or novel angiogenic therapies on tissue perfusion in PAD. Future studies will involve other novel MR methods of absolute flow quantification in PAD such as arterial spin labeling [19] and T1-mapping [20,21] as well as investigation of cuff-occlusion hyperemia as an alternative to exercise perfusion in PAD.

## Competing interests

Drs. Epstein, Meyer, and Kramer receive research support from Siemens Healthcare.

## Authors’ contributions

RSJ – Performed the imaging, analyzed the data, drafted the manuscript. AWP – Helped design the study, performed the imaging, analyzed data, drafted the manuscript. FHE – Helped design the study, co-wrote the imaging pulse sequence, and drafted and reviewed the manuscript. PFA – Co-wrote the imaging pulse sequence, analyzed the data, drafted and reviewed the manuscript. CHM – Helped design the study, and drafted and reviewed the manuscript. ALW – Designed the exercise protocol and drafted and reviewed the manuscript. DL – Analyzed data and drafted and reviewed the manuscript. JMD – Analyzed data and and drafted and reviewed the manuscript. JRH – Recruited patients and drafted and reviewed the manuscript. JMC – Performed the imaging and drafted and reviewed the manuscript. CMK – Oversaw all aspects of the study including study design, patient recruitment, imaging, data analysis, and drafted and reviewed the manuscript. All authors read and approved the final manuscript.

## Funding

Supported by National Heart Lung Blood Institute R01 HL075792 (CMK), and National Institute of Biomedical Imaging and Bioengineering T32 EB003841 (RJ, AWP).
